# The invisible frontline: experiences of public health workers during the COVID-19 pandemic and health emergencies in Colombia

**DOI:** 10.3389/fpubh.2025.1589091

**Published:** 2025-07-11

**Authors:** Sandra Martínez-Cabezas, Adriana Díaz del Castillo, Johana Linares-García, Natalia Niño-Machado, Alvaro J. Idrovo, Myriam Ruiz-Rodríguez, Catalina González-Uribe

**Affiliations:** ^1^Center for Sustainable Development Goals for Latin America and the Caribbean, Universidad de los Andes, Bogotá, Colombia; ^2^Departamento de Salud Pública, Escuela de Medicina, Universidad Industrial de Santander, Bucaramanga, Colombia

**Keywords:** health policy, health workers, health professionals, health systems, health planning

## Abstract

**Background:**

During the COVID-19 pandemic, frontline workers were widely recognized for their efforts, with an emphasis largely placed on clinical personnel providing individual care. However, public health workers, who played a critical role in managing the pandemic from a population wide perspective, received far less attention. This paper explores the experiences of public health frontline (PHF) workers in Colombia during the COVID-19 pandemic, highlighting their role in virus identification, surveillance, and guiding public health responses.

**Methods:**

Using a qualitative approach with semi-structured interviews (*n* = 83), we examine the challenges faced by the PHF, their strategies for adapting to the crisis, and the impact of the work overload they encountered.

**Results:**

The structural conditions that influenced public health responses in Colombia, shedding light on the necessity of a robust public health workforce for emergency preparedness. All the work realized to respond from a collective health perspective was performed by a PHF who felt that they were *invisible*. This invisibility had to do with the precarious working conditions that predated the pandemic, but also with a sense of being undervalued or not publicly recognized and thanked for—as opposed to clinical healthcare workers—, since public health was not necessarily considered part of the “COVID frontline.”

**Conclusion:**

The lack of a clear definition of the public health frontline during the pandemic rendered essential workers in this sector invisible, leading to less recognition compared to clinical healthcare staff and affecting their well-being, safety, and motivation.

## Introduction

Public health workers play a crucial role in the functioning of health systems, including the response to health emergencies (1–4). They detect and assess health threats, investigate events, notify, activate, and coordinate responses, collect, monitor, and analyze data to inform decision-making, communicate risk, and plan and implement vaccination programs, among others (5). A strong, well-prepared public health workforce is considered a core indicator of capacity in implementing the International Health Regulations (IHR) and indispensable for delivering the essential public health functions (4, 6, 7). Despite this pivotal role, accounts of their experiences and narratives during the recent COVID-19 pandemic remain scarce in the literature, especially when compared to the coverage of health workers who provided clinical care at the individual level. This difference is palpable in the use of the concept of frontline health workers, which was widely discussed in both global media and academic publications during the COVID-19 emergency (8–13). It was either employed indistinctly to refer both to health personnel working in clinical settings, such as physicians and nurses (8, 9, 11, 13), and to the public health workforce (14–16), or mainly used to refer to clinical staff (17–21).

In this paper, we use the term “public health frontline” (PHF) to highlight the particular roles the public health workforce plays in emergency response and the population level at which their actions take place (14). Our intention is not to downplay the importance of frontline clinical workers, but to focus on the work of the public health workforce as part of the frontline. In this term, we include decision-makers, public servants, technical OGCER, coordinators, laboratory personnel, and other staff working in public health activities. They work in areas such as epidemiological/public health surveillance, vaccination programs at national and local levels, in local health departments, private organizations, aid agencies, the Ministries of Health, and the National Institutes of Health. Their responsibilities, decisions, and actions have a broader impact compared to those of their clinical counterparts.

The limited visibility of the PHF in media and research literature might have to do with the fact that they tend to perform their work "behind the scenes" (22) and that despite its importance, the public health workforce remains a complex and often ambiguous term (4, 16, 23). It encompasses a diverse range of workers from multiple disciplinary backgrounds, including those formally trained or not, who are responsible for promoting health and preventing disease at the population level through both individual and collective services. These individuals and teams work across various organizations and sectors, including non-health sectors (4, 16, 24, 25). However, the PHF is not only overlooked in accounts of the pandemic. This lack of visibility mirrors what takes place in public policy. Public health has historically been neglected and tends to be considered of lower priority than clinical services, despite evidence of the cost-effectiveness of public health actions (1–4). Public health workers are often underpaid and have limited opportunities for professional growth and development. It is challenging to recruit and retain talented professionals, and countries face a shortage of staff. There is a lack of priority in resource allocation and a lack of political commitment to strengthening and building capacity in public health (2, 3, 24, 26).

The COVID-19 pandemic exposed these weaknesses worldwide (1, 22, 27). Since then, the Lancet Commission on Lessons for the Future emphasized the importance of effective surveillance systems and a well-trained workforce adept in outbreak investigation (28), and the World Health Organization called for greater investments in public health capacity (4). Other identified learnings include the need to strengthen public health organization, training, resources, financing, and competitive working conditions, among others (2, 16, 23, 28). Although several recommendations have been made to improve the PHF capacity, skills and competences, few publications after COVID-19 are concerned with the support and conditions that the PHF need to respond to the challenges posed by public health emergencies, especially during emergencies with a long duration such as COVID-19 (29) and to the preparation that those conditions entail (30). Moreover, the literature related to the PHF during the COVID-19 pandemic is scarce in the Latin American and Colombian contexts. Most of the research is related to healthcare workers working in hospitals (12).

In this paper, we use the multidimensional factors or domains that affected health and care workers during the COVID-19 pandemic, developed by the WHO (21). These factors include health (mental health, stress, risk of contracting the virus, and risk of death), social well-being (stigmatisation, discrimination, and care of family members), working conditions (temporary contracts, lack of psychological support), and availability and distribution (shortages, vacancies, and repurposing). Understanding the experiences and challenges of the PHF during recent emergencies, such as the pandemic, from the workers’ perspectives, could provide valuable insights into key issues to prioritize for emergency preparedness and response, as well as for the functioning of health systems.

Our aim in this paper is to describe the experiences of the PHF in responding to the COVID-19 pandemic in Colombia through a qualitative approach. We will examine their challenges, how they adapted, and the lessons that can be drawn to better support and protect the PHF before, during, and future public health emergencies. First, we will explore how the PHF organized to manage the emergency and analyze the consequences of their workload. Finally, we will propose recommendations that could be replicated in countries with similar public health infrastructures.

## Methods

### Study design

This case study, part of a national interdisciplinary project called AGORA, aimed to characterize key lessons from Colombia’s response to the COVID-19 pandemic, from the perspective of decision-makers and public health professionals who led the emergency in various regions of the country between 2020 and 2022. AGORA was designed to investigate lessons learned across diverse areas of public health during this period.

A case study was chosen as the methodology, given the role of the context in shaping the public health response to the emergency and the situations faced by the PHF. As a case study, Colombia provides a valuable example of middle-income economies that face high inequities, intra-country heterogeneities and a fragmented health system (31). Like other countries in the Americas, Colombia had a weakened public health capacity prior to the onset of the pandemic (32–35). The public health policy is formulated, coordinated and supervised by the Ministry of Health. The National Health Institute and the National Institute for Drugs and Food Surveillance execute the Ministry’s policies and focus on health research, epidemiological surveillance and disease control. Local health entities are responsible for implementing and supervising policies in their jurisdiction (36). Although the system aims for comprehensiveness in the deployment of actions, it faces challenges in coordinating with the health insurance system, a market-based system comprising diverse public and private actors. This issue falls on PHF, who must face structural problems in the development of their functions. It constitutes a typical case (37, 38).

With a qualitative approach, the study was organized into three phases: an exploratory stage in which we conducted a literature review and designed and tested the interview guidelines. The second stage comprises data collection, and the third stage data analysis.

### Participants characteristic and sampling

The selection criteria focused on identifying key actors at national, departmental, and municipal levels who participated in different roles during the COVID-19 emergency response in 2020 and 2022. Roles included senior to low-level decision-making (35), as well as managerial or technical roles in activities such as epidemiological surveillance, the Testing, Tracing, and Surveillance program, vaccination, and public health laboratories in both public and private institutions. Following a pilot test, we included international cooperation agencies due to their role in the crisis (Table 1). We conducted sampling in two phases: first, intentional sampling based on key actors' attributes (role, activity, national/local level, and region), followed by snowball sampling in different regions of Colombia.

**Table 1:** Interviewees per type of actor and gender.

Type of actor	Female	Male	Total
Public health surveillance	20	9	29
All interventions^a^	8	11	19
Laboratory	16	1	17
Testing, tracing and surveillance program	8	2	10
Vaccination program	6	2	8
Total	58	25	83

a Includes actors who could inform about more than one intervention (i.e., surveillance, testing, tracing and surveillance program, vaccination or laboratory): decision makers (*N* = 9), cooperation agencies (*N* = 6), health insurers (*N* = 2) and community leaders (*N* = 2).

### Data collection

We carried out data collection in two phases. In the first phase, we developed a map of organizations and key actors that met the inclusion criteria based on document analysis, media, and snowball referrals. A total of potential participants was identified and contacted by email or phone. Of these, 83 agreed to participate, representing a 67% participation rate, with 70% of participants being women (Table 1). The second phase involved implementing data collection (June–December 2023).

We conducted semi-structured interviews, 63 virtually via Microsoft Teams® and 20 in person. All interviews were recorded (audio or video) and conducted by at least two experienced interviewers to ensure data quality. Before each interview, participants were sent an informed consent form along with a document outlining key project details. All but one agreed to be recorded. The interviews followed an interview guide adapted to the profile of each key actor, which inquired about conditions pre-pandemic and preparation; process of adapting and implementing the response, including coordination mechanisms; barriers and facilitators; lessons learned.

### Data analysis

The information was transcribed verbatim and organized by interview type and key actor, anonymizing data by removing direct and indirect identifiers. Each transcript received an alphanumeric code, and the Nvivo® V.14 software was used.

We conducted a thematic analysis using a combination of deductive coding based on the interview guides and inductive coding (39). We initially developed 23 codes that described the context, coordination mechanisms, barriers, facilitators, and consequences on the public health staff (Table 2). These preliminary findings were discussed in interdisciplinary analysis sessions to identify patterns and salient themes and reach consensus (39). The invisibility of the PHF emerged as an overarching theme, which led to a literature review on the concept and an iterative process for refining the analysis. Three main themes were identified to organize the data: structural conditions, response, and consequences.

**Table 2:** Analysis categories.

Category	Description	Codes
Context	Structural conditions that determined the development of the health emergency and response in terms of epidemiological surveillance, the Testing, Tracing and Surveillance Program, the National Laboratory Network, and the Vaccination Program	Territorial heterogeneity
		Local health organization
		System fragmentation
Coordination mechanisms	Forms of organization implemented to face the emergency	Established mechanisms
		New mechanisms
		Intersectoral coordination
Barriers	Obstacles faced in responding to the emergency in terms of infrastructure—technology—information management, human and financial resources	Scarce personnel
		Recruitment of personnel
		Precarious labour conditions
		Work overload
		Low recognition
		Loss of institutional memory
		Lack of preparation
		Low resources
		Information systems
Facilitators	Situations, factors, resources—human-technical-technological, processes, experience that allowed the PHF to face the emergency	Leadership
		Governance
		Previous capacities
Consequences	Health, personal and professional consequences of participating in the response identified by the PHF	Stress-related illness
		Self-care
		Personal consequences
		Dealing with death
		Changes in career trajectory

Rigor criteria included credibility (triangulation of sources), consistency (detailed process description and team reflexivity), and confirmability (verbatim transcription, constant comparison of results, and analysis of limitations) (40).

## Results

### Structural conditions faced by the PHF

Structural conditions related to the Colombian health system's response to the pandemic were described in the interviews as a central element to understanding differences at the territorial level, showing inequalities in access to public health, particularly in small cities and rural areas. These regions faced challenges such as geographic dispersion, inadequate infrastructure, and limited resources. The following quote illustrates the situation:

*[…] [In this department, for example], we have many municipalities, so let's say that there is a diversity of areas, areas that are closer, areas that are far away, so let's say that in order to provide care and coverage in all public health issues, we must take into account the availability, access to health services, to public health actions, the availability of resources by the municipalities, the provider institutions that have sufficient funding to respond to events that may occur […] (Local lab coordinator)*

Local actors in dispersed areas faced budgetary limitations, resulting in precarious labor contracting and delayed access to essential resources, including supplies, transportation, and infrastructure. Interviewees attribute these challenges to the national government's staggered budget transfers to territorial institutions, which are not received at the start of the fiscal year. Consequently, municipal and departmental administrators often rely on temporary indebtedness to meet their financial needs.

*[…] the funds of the territorial institutions are allocated in this way every year, but they are disbursed in July, August, and September. During the first months, there is no real allocation of resources, but rather indebtedness. Even for the hiring of personnel, we know that unfortunately, many of these territorial institutions they are all contractors, even though they perform essential functions, which is terrible […](Technical officer-national level)*

The hiring of PHF is concerning due to its precarious nature, characterized by temporary contracts, short durations, and low pay, which can lead to labor instability and disrupted processes. Local stakeholders have reported historical instances of political interference in selection processes, resulting in appointments based on political rather than technical or merit-based criteria, which negatively impacts the efficiency and quality of public health management.

The situation for hiring PHF deteriorates with government changes, resulting in high turnover and policy shifts that disrupt continuity in human resource management. This exacerbates local authorities' challenges in securing essential public health resources at fiscal year transitions, compounding earlier issues of precarious contracts and political interference, thereby increasing inefficiencies and instability in public health management, as described by the following interviewee:

*[…] we [at the national level] had, more or less, the number of contractors needed to function. So, we were not naked, but at the territorial levels, they were naked. Why were they naked? Because in not all the territories, the joint commissions [for the transition to a new administration] authorized the hiring of personnel for December and January. […] they had to wait for the incorporation of the resources by the assembly or by the council [legislative organs] to be able to use the resources. […] many of them changed their surveillance teams, keeping in mind that in the territorial institutions the surveillance staff is minimal […] (Technical officer-national level)*

Undoubtedly, structural problems have determined the state of the PHF, which had to face COVID-19 in Colombia. Geographical dispersion, rurality, bureaucracy, and administrative processes, among other factors, determine the challenges faced in territorial planning and management.

### Responding to COVID-19 from a collective health

Responding to an emergency from the PHF entails challenges specific to the work of collective health. We will describe what this meant for the PHF in Colombia in terms of four dimensions: public health surveillance, coordination mechanisms, decision-making, and data management and analysis.

#### Public health surveillance

It is one of the cornerstones of emergency response to infectious diseases, which requires a combination of technical, technological, logistical, and human resources with 24/7 availability (59). At the beginning of 2020 in Colombia, the state of the PHF was heterogeneous across the different territories studied, due to the inequities mentioned above. Still, most shared a scarcity of personnel for basic functioning. Territories with more substantial resources, management, leadership and political will were able to increase their capacities relatively quickly, while others had to wait several months to hire new personnel. The lack of commitment or understanding of the need for a strong PHF from some mayors/governors, coupled with new local administrations, slow administrative procedures, and scarce resources, obstructed the capacity to strengthen the local PHF. The following quote illustrates administrative and human resources difficulties:

*We, the people who worked in the Ministry and the local health departments, were the same people from the beginning, because dealing with an emergency of this magnitude, which was so unexpected, clashed […] not only with the budgetary capacities, but also with the administrative management capacities […] Only in the middle of the year did we get some support staff, but there were many setbacks. (Technical officer-national level)*

This meant that the scarce PHF took on new tasks in addition to routine activities and worked long hours to cover 24/7 shifts and deal with an unprecedented volume of surveillance cases; some worked without a legal contract for months. Moreover, they had no time to recharge between waves, since when infections declined, they resumed responsibilities they had had to postpone while responding to COVID-19.

*[…] I tell you, the shifts started at 7:00 a.m., but we didn't know what time we would get off. (Local lab coordinator)*

All of this caused obstacles in the primary processes designed for collective health. Key surveillance processes were delayed or hindered (e.g., untimely diagnosis and control, insufficient case analysis and reporting), and the PHF was overburdened from the start. Personnel turnover continued throughout the pandemic, which generated the need for continuous training, further delays, and additional wearing of staff, as illustrated by the following quote:

*If it wasn't that they didn't have people, it would be the turnover of human talent. Then the human talent rotated so much that the processes were reversed, because you had already trained a lot of people, you had already explained to them, and you went backwards… (Technical officer-national level)*

Territorial inequities meant that some PHF teams dealt with structural barriers for which they had few resources and sometimes had to solve on their own (i.e. purchase of equipment or internet access). Limited telephone networks meant that telephone tracing was not possible in some municipalities, while field teams were exposed to a lack/uncertainty of transport availability to remote areas and scarce food during fieldwork. In many places, accepting these working conditions was seen as a sign of “vocation” or commitment.

#### Coordination mechanisms

The PHF organized the response through established coordination mechanisms and new strategies according to the IHR, the National Risk Management System, and guidelines from the National Institute of Health (i.e., unified command posts, crisis/situation rooms, risk analysis rooms, incident command, and cross-border surveillance). New mechanisms were devised for managing personnel and coordinating with other actors (i.e. management cells based on agile methodologies with 24-h response goals, intersectoral committees, experts/advisors roundtables, technical support for territories). All this could have yielded positive results in terms of efficiency, decision-making, and coordination, but also meant an overload for the PHF, which participated in several of these mechanisms, ranging from several times per day to daily, biweekly, or weekly, or with 24/7 availability. The following quote illustrates this overwork:

*Because one thing is that it sounds wonderful that the cellphones were working, and you're meeting every day at 6:00 a.m. and making decisions every 24 hours, that sounds great. But go and do it and put up with that pace for a week, we were all sick already. (Local decision maker)*

#### Data management and analysis

Other crucial activities of the PHF included daily registration, confirmation, and monitoring of data. Just as their clinical colleagues faced an exponential growth in patients and overcrowded hospitals, the PHF dealt with amounts of data that continued to grow exponentially, coupled with overflowing and collapsing information systems that had limited infrastructure and human capacities. Their work was also time-sensitive. They had to prepare daily reports for decision-making purposes, which were also broadcast in mass media and reported to citizens on various platforms. In remote or small territories, internet connection difficulties, coupled with insufficient information systems, implied a more manual workload, at times resulting in lost work or having to work throughout the night to upload information that needed to be ready at very early hours.

Thus, the feeling of work overload for the PHF might have been mainly due not only to limited resources, but also to the exacerbated production of data. Without a doubt, in times of long-duration emergencies, it is exhausting to respond to the increased need for data to control the disease, guide public health decision-making, respond to control agencies, and communicate risk, among other tasks.

*[…] Our team did not grow; we were only two people. […] We updated the daily data in Power BI, which was reported daily by the mayor. […] We worked until 1-2 am. I would send indicators to the press, minimum indicators that had to be kept, and data requested by the Attorney General's Office. The day was not over until the data were updated. […] we said that we could not go on like that, we were exhausted […] (Local technical officer)*

#### Decision-making

Working at the PHF entails making decisions that affect populations. During the COVID-19 pandemic, these decisions were made under intensified pressure, uncertainty, and public scrutiny. Public servants ought to respond to increasing amounts of requirements from control agencies and citizens, keep up to date with constantly evolving evidence, policies and regulations, and respond to inquiries and criticisms from different stakeholders, to name a few. These demands interfered with their ability to perform their work, while also increasing their stress levels. The following quote illustrates what PHF faced with the control institutions:

*Another thing that affected us a lot and that sometimes was very much against everything we did, was all the vigilance of the control institutions [Attorney General's Office or Prosecutor's Office]. You really cannot imagine what we suffered when we were planning […] the control institutions did not understand that you had human errors, that there were losses [of vaccines] as the world had […] So, in a pandemic, responding to around 22,000 petitions, requirements of the control institutions and the citizenry, and at the same time working to respond to a whole country, well, there is no explanation for that. (Technical officer-national level)*

Also, some interviewees felt that previous experience was not necessarily considered in decision making, and more experienced personnel had to “justify” that their experience in responding to epidemics was valuable (at times where timely response was critical), as described by the following quote. Other times, the overload did not allow for the transfer of knowledge to the newly hired personnel. All this speaks to a lack of knowledge management strategies or institutional cultures to support it, which in turn affect the work of the PHF.

*[…] part of the discussion we were constantly having was precisely that we had to take advantage of that experience [of AH1N1], and sometimes it was not so easy for some to recognize that experience […] I [worked on AH1N1] and I told them, "We did this, look, the drill says this, this has to be done". And some people said, "no, no, that's not like that". Fifteen days later, they said, "Yes, there was a mistake here, it is like [what you said]". (Local decision maker)*

Finally, working with collective health also meant that the PHF dealt with experiences of loss and death in their personal and professional lives, but also in the communities and populations they were responsible for. They dealt with death not only in the private sphere of their work but also with illness and deaths that they were made accountable for publicly and legally. Most of the interviewees addressed this issue using their own coping mechanisms without organizational support. A technical OGCER described one of these situations:

*She is now the Secretary of Health of R. […] I called her to ask how they were doing, and she was sobbing, crying her eyes out. She told me, "They are dying, they are dying on me, and I can't do anything more". (Technical officer, national level)*

In summary, the response to the emergency in Colombia revealed significant challenges inherent to public health work. The combination of limited resources, high staff turnover, and political dynamics created considerable pressure on workers, who were forced to adapt quickly while facing high workloads. During the crisis, long-standing structural problems were revealed that require urgent attention to improve the effectiveness of responses in future emergencies, to ensure that public health professionals have greater support and stability.

### Public health workers: the invisible frontline

All the work described above was performed by a PHF who felt *invisible*. This invisibility was partly due to the precarious working conditions that predated the pandemic, but also to a sense of being undervalued or not publicly recognized and thanked, unlike their clinical healthcare counterparts, as public health was not necessarily considered part of the “COVID frontline”. This perception was felt even among decision-makers who were placed in the spotlight of media or other stakeholders’ attention, as public accountability did not necessarily mean recognition, being valued, or support. The following quotes illustrate the invisibility of the PHF:

*The health Secretariat said, “Of course, everyone celebrated the doctors, but the health secretariats were never recognized”. (Cooperation agency OGCER)*

*Obviously, hats off to all the clinical frontline, but look, I questioned why the decree that gave money for that health care frontline did not include us. We were so invisible even to ourselves, because that decree came out of the [health sector] (Technical officer national level)*

This invisibility meant that some PHF teams did not receive sufficient protection equipment, vaccines (i.e., for workers in cooperation agencies), or institutional support to cope with the personal, professional, and health consequences of working on the frontline. Most organizations were not prepared to deal with these consequences, so they remained completely or partially unattended. Some interviewees, such as the following OGCER, felt that during the pandemic, they were not seen as human beings and their needs were neglected:

*[…] I believe that, psychologically speaking, the organization has also not handled this issue effectively. What impact does the entire pandemic have on the population and public servants? Not only because of the pandemic, but also due to all the additional work we had to do. (Technical officer, national level)*

*[…] it was so much in such a short time. Living with the fact that I was far away from my mom, and I didn't know what was going to happen with her [sobs]. […] And nobody asked me about that. We are human beings, too. And they only saw organizations; they didn't see human beings. (Local decision maker)*

Given this lack of institutional resources, some leaders tried to create supportive environments with strategies that ranged from guaranteeing food/coffee, to shifting roles to allow for rest, allowing time to “disconnect”, and advocating for their teams to be considered frontline respondents and thus included in the monetary incentives that were offered. However, because this response was not planned or institutionalized, these mechanisms relied on specific individuals, were reactive, and were seldom reported. In some cases, this created inequalities, as some PHFs received incentives (e.g., surveillance), while others did not (e.g., lab personnel) and thus remained invisible. A cooperation agency OGCER described what this entailed:

*[…] For me, it was a challenge to lead teams in the virtual world and to manage the mental health of my team. It was very complex to measure the times, to try not to be connected 24-7, to have time for self-care, to work with people who suddenly had anxiety, depression, in the middle of the confinement and, even so, to do all these things of the response, all we had to do at work […] And to do it a little alone, right? Because nobody knew how to do it either, there was no accompaniment, so it was a matter of learning by doing. (Cooperation agency officer)*

Also, some PHFs described having been left alone in their efforts to respond to the emergency. In some territories, this was due to a lack of intersectoral collaboration. Others, as in the following quote, described situations in which although they could have had support from other areas, some of them chose not to support the PHF and prioritized their safety and that of their families:

*[…] It was such a significant challenge that there was a moment when I was exhausted and couldn't take it anymore. I went into a horrible, horrible crisis of anxiety and depression, because of the pressure I was under to see that everyone was calling* me*. The phone would ring at 1:00 a.m., 2:00 a.m., and I would get up again at 6:00 a.m. and the phone would ring all day long, asking me for an ambulance, for a certificate, […] that there were investigations, that there were follow-ups… […] and when something did not work, everything came back to you. It was quite stressful, all that process. The truth is that's when I realized not everybody was in – not everybody wanted to help with the pandemic because they wanted to safeguard their own lives and their families' lives. They had a responsibility to support, but they didn't want to do it. Many said no and no […] (Local technical officer)*

### Consequences of working on the collective frontline

#### Health and well-being

Just as with the clinical frontline, the PHF experienced health consequences of responding to the pandemic. They also had a higher risk of infection, got sick, and some lost their lives to COVID-19. Physical health was also affected by the long working hours and the stress. Some interviewees reported they suffered from physical pain, worsening of chronic conditions, or new diagnosis, such as the decision maker in the following quote. Some of them decided to retire or request a change in job roles during the pandemic because of these consequences.

*Some of us are very sick, most of us, including myself, we have finished responding to the pandemic, and most of us are at this moment… […] in programs for chronic conditions. We all came out with hypertension, some of us with diabetes (Local decision maker)*

The PHF also experienced burnout, depression, and anxiety that even two years later still affected their lives. This was related to the work overload, but also to conditions particular to their work in collective health, such as the stress derived from the responsibility of responding to an emergency of such scale and speed, making population decision with high uncertainty, having their work on the spotlight, and the pressure and attention from all sectors (media, citizenry, control institutions).

*[…] that's when I said “no more”, because I was getting sick, because of the stress I was already carrying […] I was physically exhausted, and that was when I asked for a transfer, and then I left […] I started to somatize, feeling physically tired and becoming irritable. And of course, it was already due to the exhaustion I was feeling because of the work we were already doing […] I said – “No, no more, I mean, I'm not for this, I don't have to do it, I've already accomplished what I was assigned to do” – (Local technical officer).*

While dealing with all this, they felt insufficiently prepared to care for themselves and their colleagues. In a few cases, organizational and individual strategies were implemented, such as the establishment of psychosocial support teams or wellness activities (e.g., concerts, spaces for relaxation, and improvements to common areas). Yet, interviewees such as the following decision maker considered that these were insufficient, given their actual needs or the lack of enough time to participate while responding to the emergency:

*[…] the [organization] generated mechanisms for the care of health personnel. So, we organized concerts, we created mental health support teams that were mobilized 24 7. […] but the truth is that it is still complex, because the times have not been correct. […] there was never a pause to say, "come on, we are going to give you a month to rest", no. Most of us lasted two and a half years without a break. We had our vacations cancelled, we had Easter [vacation] cancelled, we had December [vacation] cancelled, everything. (Local decision maker)*

The importance of acknowledging these consequences for the PHF and the lack of support there is, is that they need to be considered to improve emergency preparedness. Interviewees highlighted the need to care for the PHF as one of the key lessons learned from COVID-19 and one of the necessary changes for future epidemics:

*This is key in emergency preparedness because if it is not foreseen and planned, it is overlooked. The importance of taking care of human resources is underestimated in emergency preparedness. Look, we prepare for emergencies, but we don't prepare for the people. […] Institutionally we are little machines. All the time we think of emergencies, but it never occurred to me personally to think of myself in an emergency. It never occurred to me. […] So yes, the human [aspect] matters, and part of the pandemic preparedness is us and we don't think about it. That's missing. That wasn't thought about or planned for. (Technical officer-national level)*

#### Trajectory change and loss of institutional memory

Working at the PHF during the pandemic and the health consequences that it entailed caused two important issues. First, the decision of several workers to change their professional trajectory. This change is associated with a need to seek personal well-being. The impact of having been part of the invisible frontline led many to reject the idea of returning to similar positions in the future, as expressed in the next quote. Particularly, key actors expressed feelings of frustration and disillusionment because of the problems they faced and the lack of recognition they felt. It is important to mention that, although some people sought this change, others did not have their employment contracts renewed when the intensity of the emergency decreased.

*Interviewer: What did it mean to you to have been part of that frontline?*

*Interviewee: Nothing, just problems. [From the] Comptroller's Office, Attorney General's Office, Prosecutor's Office […] I never want to occupy a position like that again in my life […] (Local decision maker)*

Second, this change has medium- and long-term implications for future emergencies, given the loss of institutional memory. Although today there are infrastructural improvements in the territories, the difficulties in retaining experienced personnel lead to a lack of institutional progress, given the loss of tacit knowledge and the capacity to learn from mistakes and successes. No doubt, this could impact the management of future emergencies and constitutes a great challenge in the management of the health workforce.

#### Personal lives

In addition to their work, the PHF also dealt with personal situations derived from the emergency that exacerbated or increased the mental health consequences of work stress: the social rejection to health personnel generated by the fear of contagion, their own fear of infecting family members, the difficulties or impossibility of seeing children or parents that lasted months, and the feeling of neglecting their families or themselves while working long hours. This was especially noteworthy for workers who were also caregivers. Some interviewees felt that during the pandemic they were not seen as human beings and their personal and health needs were neglected by other people and organizations.

*I neglected my son a lot and I also distanced myself from him a lot because I dedicated myself to work. […] I would arrive at his school crying for help with the child, I saw that he was not well and I could not help him […] because of the pandemic I neglected my home and I neglected myself mentally too, because imagine, I ended up in a state of crisis, of anxiety, which I am still treating and it was because of work stress (Local technical officer).*

## Discussion

This study described the experiences of the workforce that constituted the backbone of the population response within the Colombian health system during the COVID-19 pandemic. These experiences are closely associated with structural elements of the health system, particularly its fragmentation and weaknesses in health governance at the territorial level, which have manifested in delayed hiring, a multiplicity of tasks, and significant impacts on the health, social and well-being, working conditions of PHF workers (21). In the medium and long term, these issues resulted in a loss of institutional memory, which is invaluable for managing future health emergencies.

Despite these structural limitations, territorial heterogeneity, and inequities, Colombia emerged as the country with the most effective response to the pandemic in Latin America, according to Bloomberg's (41) resilience ranking. This success can largely be attributed to the overexertion and overload of the PHF and other health workers. Many of the challenges faced by the workforce predated 2020, but the pandemic highlighted that, if the PHF was already under-resourced and understaffed before COVID-19, their ability to respond adequately to such an emergency became even more constrained. This situation was not unique to Colombia; it was shared by multiple countries regardless of their health systems, governance structures, and resources, both in the global north and south, and within and between countries (1–3, 14, 22, 42).

These conditions, combined with the established and newly implemented mechanisms for coordinating the response, resulted in increased work overload, which, given the prolonged duration of the pandemic, led to burnout and exhaustion among PHF workers. This burden negatively affected the physical and mental health, well-being, and working conditions of the PHF, which converges with the multidimensional factors proposed by the WHO, indicating that they apply not only to health and care workers but also to the PHF (21).The majority of interviewees highlighted these three factors or areas, as evidenced in the findings. The PHF faced these challenges without adequate resources or strategies to care for themselves and their teams, and they reported feeling undervalued, dehumanized, and made invisible, despite their critical role in managing the pandemic from a population perspective. They reported a few strategies for adapting to these conditions, which were mainly attributed to leadership initiatives aimed at protecting their staff. This aligns with the interviewees’ accounts of being overburdened. Some appealed to a sense of vocation while dealing with the situation. Although this spirit might have helped motivate personnel during such difficult times, it also placed the responsibility on workers while hiding structural problems for which they should not be responsible.

Similar perceptions and experiences have been documented by authors in other countries Studies have shown that during the pandemic, PHF experienced high levels of burnout, mental health problems, threats, and a pervasive sense of being misunderstood and undervalued compared to their healthcare colleagues (16, 43–45). A study conducted in the United States reported that 13.6% of public health personnel experienced poor physical health for at least 14 out of the last 30 days, while 41.4% reported similar issues regarding mental health. Furthermore, a significant percentage of respondents indicated plans to leave their positions or retire (16).

The multidimensional factors affecting the PHF had detrimental consequences for emergency preparedness and response, resulting in the invisibilization of these personnel. It compromised the quality and timeliness of the response, but also compromises the capacity to recover from the emergency and to respond to future emergencies, given that some of these workers already left the field of public health, are not interested in roles of decision making anymore, or are not willing to work in the PHF in future emergencies. Evidence from the USA, China, and Taiwan also showed an increased tendency for the personnel to change career paths after the pandemic (44, 60, 61). In our study, all of this was due to the health and personal/professional consequences they faced or continue facing, even 2 years after the pandemic ended.

The invisibility of the PHF can be explained from at least two perspectives. First, a health model perspective. Current health systems are based on a curative model centered on individual care. During the emergency, the weakness of this system became evident, given the historical and social value of public health for nations, who faced the challenge of moving from an individual approach to a collective and community approach based on the prevention of the spread of the virus, under principles of "effectiveness, universality, solidarity, integrality, unity and participation" (46), with limited resources, diminished capacities and labor precariousness of workers in the sector. The response was heterogeneous given the installed baseline capacity, territorial leadership and the level of sectoral and inter-sectoral coordination. While some were quick to take up the response, others lagged behind (47). These territorial differences are one of the main barriers that the country has not overcome after the reform. Public health thus represents a political and governance challenge, where it is necessary to understand the barriers and possibilities for inter-sectoral and intra-sectoral coordination, as well as to ensure adequate investment to maintain public health infrastructure and personnel capacities. In addition, strong leadership is needed to ensure that public health policies are sustainable and can withstand the shifts in power and economic fluctuations that countries face.

Second, it can also be understood from a gender perspective. Within emergency responses to COVID-19, public health roles have received less visibility and have historically had precarious working conditions worldwide (1, 3, 16). This has to do with a discipline/profession that is feminized, women seem more likely to be in charge of caring for others, especially demanding tasks in the context of a pandemic (48). It is noteworthy that 70% of our interviewees were women who worked as part of the PHF in the territories. As mentioned in the results, the triple burden was evident in those women who, in addition to fulfilling their work duties at extreme hours, had to take care of their families and themselves at the same time. However, we clarify that gender was not a category of analysis and here we only intend to show an element for future research given that the composition of health workers in Colombia is 78% women (49). The health crisis generated by COVID-19 highlighted the need to continue to reflect on the gender inequalities faced by women in the health sector (50). This is a clearly structural issue, a, as in other epidemics such as Ebola, women have occupied care-related roles and there is an underrepresentation of women leaders in global health (51)

During the pandemic, several publications focused on identifying risk factors associated with sex (52) and on the roles of gender and frontline healthcare workers (53). In future epidemics and long-duration emergencies, it is essential to reassess the care that women public health workers receive, in order to prevent contributing to the widening of gender gaps within this group of workers.

The consequences faced by the PHF are worrisome in human terms and raise questions about the lack of capacities of emergency response systems to care for their workforce and the human cost of epidemics that are not accounted for. It also raises practical concerns, as there are no robust knowledge management strategies in place to safeguard the PHF’s tacit knowledge and experiences from being lost. The skills, competences, and willingness of the personnel to respond are critical for emergency preparation and resilience, and the workforce is one of the core capacities of the IHR (4, 7, 54–56).

Through this research, we identified a gap in the term “frontline”, which tends to relate to those who oversee individual patient care, rather than those who are in charge of care at a population level. The latter can also include non-health workers (i.e., social scientists, developers, data managers, lawyers, and communication professionals). It is striking that, while in some contexts public health workers are visible as part of the frontline emergency response workforce (14, 44) in others they are excluded from accounts of health workers responding to COVID-19 (21). Some WHO reports did not specifically include the impact on public health personnel as part of the frontline responders (18, 21).

Therefore, we believe that in times of crisis, such as the COVID-19 pandemic and epidemics in general, these two primary lines of care (individual and collective) must be integrated to protect staff working in the response, regardless of the type of work they perform.

### Recommendations for future preparedness

Protecting the PHF is essential for maintaining robust public health systems and establishing and maintaining basic capacities for surveillance and response, as well as complying with the legally binding IHR (5). This requires explicitly incorporating personnel support into emergency preparedness plans, addressing health challenges and lives with minimal disruption, and fostering resilience while meeting urgent demands. As Ferrinho et al. (57) argued, balancing medium—to long-term approaches that allow for preparedness and resilience with the needs of urgent requirements is not an easy task. However, it is possible to plan for all of this once it is appropriately acknowledged and given weight (58).

Key strategies include improving labor welfare to combat the sector's historical precariousness, enhancing job stability, and ensuring safe working environments to minimize risks such as contagion. Emotional and psychological support must also be prioritized to help manage stress, alongside respecting rest periods even during prolonged emergencies like COVID-19. In addition, the contributions of the PHF must be recognized both publicly and institutionally, with robust political leadership driving systemic changes. This involves balancing individual and population health needs, managing human talent effectively, and ensuring generational continuity through knowledge management and mentorship programs.

### Limitations

This study had several limitations to consider. Firstly, burnout experiences meant that several people who worked during the emergency were no longer part of the same institutions. In addition, time constraints, especially for decision-makers, led the team to increase the number of potential interview contacts and to use snowball sampling to increase potential uptake. This means that the collected experiences may not include the accounts of other workers who were not identified or contacted. It is not possible to establish how their experiences differ or resemble those actually gathered. Secondly, given the time elapsed at the time of the interviews and the intensity of the experienced events, some participants had difficulties in remembering circumstances. Therefore, we focused the interviews not on specific facts but on how the interviewed made sense of and reflected on the events at the present time. Third, some interviewees were concerned that discussing their work during the emergency would lead to criticism or evaluation of their work performance. We emphasized the confidentiality of the data and made specific efforts to protect their privacy. Yet it is possible that we were unable to collect some experiences, especially the more intense or difficult ones to speak about. However, we identified comparable experiences in our literature review, which suggests that we were still able to gather relevant information despite this. Finally, most of the interviews were conducted online. Although challenging in terms of trust and interaction, they met the objectives and fostered positive interactions. Observations and ethnographic methods were not conducted due to the retrospective nature of the experiences analyzed.

## Conclusion

The lack of a clear definition of the public health frontline during the pandemic rendered essential workers in this sector invisible, resulting in less recognition compared to clinical healthcare staff and negatively impacting their well-being, safety, and motivation. Precarious labor conditions, such as temporary contracts, low salaries, and insufficient resources, hindered their effectiveness. Additionally, instability in hiring during government transitions led to high staff turnover and disrupted the pandemic response. The COVID-19 crisis highlighted the urgent need to define and recognize the role of PHF in emergency planning, ensuring adequate support, resources, and institutional backing to integrate all frontline workers and enable a coordinated, effective response in future emergencies.

## Data Availability

The datasets presented in this article are not readily available because this project used qualitative information. Requests to access the datasets should be directed to SM-C, sp.martinez@uniandes.edu.co.
